# Portomesenteric Venous Thrombosis after Bariatric Surgery: A Case Series and Systematic Review Comparing LSG and LRYGB

**DOI:** 10.3390/jpm14070722

**Published:** 2024-07-04

**Authors:** Raquel Gomes, André Costa-Pinho, Francisca Ramalho-Vasconcelos, Bernardo Sousa-Pinto, Hugo Santos-Sousa, Fernando Resende, John Preto, Eduardo Lima-da-Costa, CRI-O Group

**Affiliations:** 1 Faculty of Medicine, University of Porto, 4200-319 Porto, Portugal; up201807501@up.pt (R.G.);; 2 Obesity Integrated Responsibility Unit (CRI-O), São João Local Health Unit, 4200-319 Porto, Portugal; 3 MEDCIDS—Department of Community Medicine, Information and Health Decision Sciences, Faculty of Medicine, University of Porto, 4200-319 Porto, Portugal; 4 CINTESIS—Centre for Health Technologies and Services Research, University of Porto, 4200-319 Porto, Portugal

**Keywords:** bariatric surgery, laparoscopic sleeve gastrectomy, laparoscopic Roux-en-Y gastric bypass, venous thromboembolism, portomesenteric venous thrombosis

## Abstract

(1) Background: Portomesenteric Venous Thrombosis (PMVT) is a rare but serious complication of Metabolic Bariatric Surgery (MBS). Although more frequently reported after laparoscopic sleeve gastrectomy (LSG), the risk factors for PMVT remain unclear. This study aims to compare the incidence and determinants of PMVT between LSG and laparoscopic Roux-en-Y gastric bypass (LRYGB). (2) Methods: A retrospective analysis of 5235 MBSs conducted at our institution between 2015 and 2023 identified five cases of PMVT. Additionally, a systematic review in March 2023, covering PubMed, Web of Science and Scopus, was performed. Several data were analyzed regarding risk factors. (3) Results: In our case series, the incidence of PMVT was 0.1%. The five cases described involved four females with a BMI between 39.7 and 56.0 kg/m^2^. Their comorbidities were associated with metabolic syndrome, all women used oral contraceptive and two patients were diagnosed with thrombophilia or pulmonary embolism. Per protocol, thromboprophylaxis was administered to all patients. Diagnosis was made at a median of 16 days post-surgery, with abdominal pain being the main presenting symptom. Acute cases were managed with enoxaparin, unfractionated heparin and fibrinolysis. One patient required surgery. Ten studies were included in the systematic review and 205 patients with PMVT were identified: 193 (94.1%) post-LSG and 12 post-LRYGB. The most common comorbidities were dyslipidemia, hypertension, diabetes, sleep apnea and liver disorders; (4) Conclusions: PMVT is a potentially life-threatening complication after MBS, requiring preventive measures, timely diagnosis and several treatments. Our findings suggest a higher occurrence in women with an elevated BMI and post-LSG. Tailored thromboprophylaxis for MBS patients at risk of PMVT may be warranted.

## 1. Introduction

Obesity is defined as excessive fat accumulation with a body mass index (BMI) equal to or exceeding 30 kg/m^2^. It is a significant global health concern with increasing prevalence. In 2022, 43% of adults aged 18 and above were overweight and 16% were obese [1]. Obesity is associated with an elevated risk of mortality and various chronic conditions, including diabetes, cardiovascular diseases, gastro-esophageal reflux disease, musculoskeletal pain, depression and several forms of cancer [2,3].

Metabolic bariatric surgery (MBS) has emerged as a viable treatment for individuals with a BMI ≥ 35 kg/m^2^ regardless of their obesity-related comorbidities, offering sustainable long-term results. Laparoscopic Roux-en-Y gastric bypass (LRYGB) and laparoscopic sleeve gastrectomy (LSG) are the predominant procedures, accounting for approximately 90% of surgeries [4]. These surgeries have a high safety profile and are increasingly performed worldwide to address the obesity epidemic, yielding consistent and favorable short and long-term outcomes.

Severe obesity and laparoscopic surgery are recognized risk factors that increase the risk of venous thromboembolism (VTE), including deep vein thrombosis (DVT) and pulmonary embolism (PE). Consequently, patients undergoing MBS face a moderate to high risk of VTE, which is further exacerbated by their comorbidities. Other high-risk comorbidities include venous stasis, BMI ≥ 60 kg/m^2^, truncal obesity and sleep apnea [5].

A severe complication of MBS is portomesenteric venous thrombosis (PMVT), a rare life-threatening event that involves the portal vein (PV), its intrahepatic branches and potentially extends to the superior mesenteric vein (SMV) and splenic vein (SV). PMVT is associated with intra-abdominal inflammatory conditions that cause injury to the portal system [6]. A recent systematic review reported that the PMVT incidence after MBS is 0.419% and that the mortality is 1.33% [7], although other studies suggest higher mortality rates (3.6%) [8]. The risk factors for PMVT remain unclear, although factors such as the surgical technique, the patient’s comorbidities and their thrombophilia history likely contribute to increased risk [9].

The perioperative thrombophylactic guidelines for patients needing MBS are not well established. The European Society of Anesthesiology (ESA) [10] and the American College of Chest Physicians (ACCP) [11,12] recommend mechanical prophylaxis with intermittent pneumatic compression (ICP) during and after bariatric procedures, along with anticoagulation therapy, preferably using low-molecular-weight heparin (LMWH). General thromboprophylaxis measures such as early ambulation and optimal hydration are also recommended [10]. However, considering the potential differences in pathophysiology, it remains unclear whether these recommendations effectively prevent PMVT, as they are primarily aimed at VTE prevention.

Patients with PMVT typically present with diffuse abdominal pain, nausea, vomiting, leukocytosis and fever. The gold-standard exam for diagnosis is abdominal contrast-enhanced computer tomography (CT). Pharmacologic anticoagulation with LMWH, unfractionated heparin (UFH) or vitamin K antagonist is the preferred initial treatment, but further treatments such as fibrinolysis through percutaneous vascular access or surgery may be warranted [8].

This study was prompted by a critical case of PMVT observed in our hospital in 2022. This event prompted us to further research similar cases. We analyzed all patients with PMVT after MBS in our institution and conducted a systematic literature review. The primary objective was to compare the incidence, patient characteristics and risk factors for PMVT after LSG and LRYGB.

## 2. Materials and Methods

A retrospective analysis of all MBS procedures performed in a single high-volume center between 2015 and 2023 was conducted to identify patients diagnosed with PMVT. A total of 5235 surgeries were performed and five cases of PMVT were identified.

### 2.1. Search Strategy

The systematic review was conducted according to the Preferred Reporting Items for Systematic Reviews and Meta-Analyses (PRISMA) guidelines statement [13]. A comprehensive electronic literature search was performed on PubMed, Web of Science and Scopus on 9 March 2023. The search items are listed in the Supplementary Information. A manual screening of the list of references regarding relevant papers was also performed to supplement the literature search.

### 2.2. Study Selection

The study selection was composed of two phases. In the screening phase, two authors (RG, FRV) independently screened the abstracts obtained from the database search. Afterwards, the full texts of potentially relevant articles were retrieved for further assessment and disagreements were resolved by consensus-based discussion or by consultation with a third reviewer (ACP).

### 2.3. Eligibility Criteria

The inclusion criteria were studies reporting thrombosis in any branch of the portomesenteric–splenic axis after LSG and LRYGB in adults (18–65 years). The exclusion criteria were studies not reporting PMVT, robotic surgery and open surgery (laparotomy). Meeting abstracts, commentaries, editorials, letters, case reports, systematic reviews and meta-analysis and animal studies were also excluded.

### 2.4. Data Extraction

Relevant data were extracted by two independent authors, and conflicts were resolved by consensus. We extracted available data regarding the study characteristics (authors name, year of publication, study period, sample size, type of study and country), patient characteristics (age, sex), BMI, history of thrombosis and/or thrombophilia, comorbidities (diabetes, hypertension, dyslipidemia, sleep apnea, liver disorders), oral contraceptive use, type of surgery, operative time, length of hospital stay, antithrombotic prophylaxis and characteristics of PMVT (time until event, symptoms and type of treatment).

### 2.5. Quality Assessment

A methodological quality assessment of the included studies was performed independently by two authors (RG and FRV) and disagreements were resolved by consensus or by consultation with a third reviewer (ACP). The included studies were assessed using the NHLBI quality assessment tool [14].

## 3. Results

### 3.1. Case Series

Our case series of patients with PMVT after MBS is summarized in Table 1. As per the protocol, all patients underwent evaluation by a multidisciplinary team preoperatively and had a minimum follow-up period of 3 years. Thromboprophylaxis included ICP during surgery, as well as compression stockings, before and 24 h after surgery. Fast-track protocols involving early mobilization and early oral hydration were implemented. Additionally, all patients received chemoprophylaxis with LMWH (Enoxaparin 40–60 mg) the day before surgery, continuing for 2 weeks for cases up to 2022 and 4 weeks thereafter, following a change in the hospital’s prophylaxis protocol after Case 4.

#### 3.1.1. Case 1

A 43-year-old female with a BMI of 40.4 kg/m^2^ underwent LSG in August 2015. Eleven days post-LSG, she presented to the emergency room (ER) with diffuse abdominal pain and fever. She had no relevant comorbidities and was taking oral contraceptive (OC). An abdominal CT scan revealed complete thrombosis of the SMV and partial thrombosis of the PV. She was treated with Enoxaparin 100 mg twice daily, with favorable clinical evolution and discharged home after twelve days. A factor II G20210A heterozygote gene mutation was detected and long-term anticoagulation with Warfarin 5 mg was recommended.

#### 3.1.2. Case 2

A 23-year-old female with a BMI of 45.9 kg/m^2^ underwent LSG in December 2016. Thirteen days post-LSG, she presented to the ER with abdominal pain, vomiting and hematochezia. She had diabetes, which was treated with metformin, asthma, and was on OC. An abdominal CT scan demonstrated extensive thrombosis involving the SMV, PV and SV. She was hospitalized and received treatment with UFH 1980 UI/h, guided by the activated partial thromboplastin time (aPTT). She developed severe hematemesis, leading to the discontinuation of anticoagulation the following day. Despite best medical treatment, her condition evolved rapidly with multiple organ failure, and the patient died three days later.

#### 3.1.3. Case 3

A 42-year-old male with a BMI of 39.7 kg/m^2^ and hypertension (HTN), dyslipidemia and sleep apnea underwent LSG in November 2019. Nine days after surgery, the patient developed PE and was treated with Enoxaparin 100 mg. After discharge, he maintained anticoagulation with Warfarin 5 mg for three months. In August 2021, nearly two years later, an abdominal CT performed for unrelated reasons revealed thrombosis of the PV and SV. The patient was referred for an immuno-hemotherapy consultation: as PMVT was asymptomatic, no treatment was instituted. The thrombophilia study was negative and thromboprophylaxis was recommended before a high-risk situation occurred. Warfarin was not maintained due to the absence of identifiable risk factors other than the risk associated with the surgery.

#### 3.1.4. Case 4

A 23-year-old female with a BMI of 48.2 kg/m^2^ underwent LSG in July 2022. Sixteen days post-LSG, she presented to the ER with abdominal pain, fever, nausea and vomiting. She had sleep apnea and was on OC. Abdominal CT led to a diagnosis of complete thrombosis of the SMV and partial occlusion of the PV. Despite optimal treatment with UFH, there was progression to intestinal ischemia, requiring surgical intervention with bowel resection twenty-five days post-LSG. The patient’s hospitalization extended to almost one year and was marked by multiple complications, including infectious, pulmonary and nutritional issues. Dehiscence of the LSG staple line was detected three months post-LSG. More than one year after, the patient died because of a gastro-pleural fistula that could not be controlled by several treatments.

#### 3.1.5. Case 5

A 32-year-old female with a BMI of 56.0 kg/m^2^ and a history of polycystic ovarian syndrome treated with OC underwent LSG in June 2023. Thirty-nine days post-LSG, she presented to the ER with abdominal pain and nausea. Abdominal CT revealed complete thrombosis of the SMV and partial thrombosis of the PV. Initial treatment included UFH (adjusted to aPTT, administered for five days), followed by fibrinolysis using alteplase (0.5 mg/h) and UFH 500 UI for two days and then 1 mg/h of alteplase for another three days. The patient showed a favorable clinical evolution and was discharged after twenty-three days on Warfarin 5 mg. The thrombophilia study yielded negative results.

### 3.2. Systematic Literature Review

**Figure 1:**
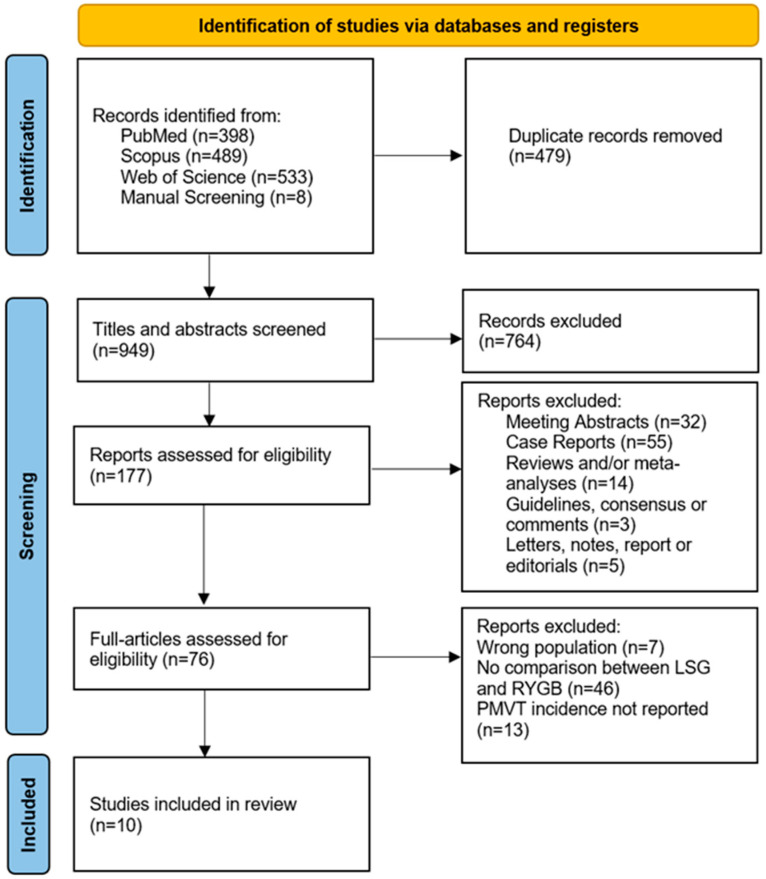
PRISMA flow diagram.

The systematic review yielded a total of 941 studies: 398 from PubMed, 489 from Scopus and 533 from Web of Science. After assessing the full texts for eligibility, 10 studies were included [15,16,17,18,19,20,21,22,23,24,25]. The PRISMA flowchart is shown in Figure 1. The main reasons for exclusion were studies lacking a comparative analysis between LSG and LRYGB, the omission of PMVT incidence and the inclusion of study populations not meeting the predefined inclusion criteria.

The NHLBI quality assessment tool results are displayed in Supplementary Materials Tables S2–S4.

The included studies, published between 2012 and 2022, comprised seven retrospective (six cohorts and one case-control study), one prospective cohort study, one cross-sectional study and one case series (Table 2). Despite documenting PMVT incidence following LSG and LRYGB, most studies lacked comprehensive information on patients’ characteristics and risk factors associated with each surgical procedure.

The patient characteristics and other variables studied are presented in Table 3. Among the 104,867 patients undergoing MBS, LSG was the most frequently performed procedure, accounting for 69,338 (66.1%) cases. PMVT occurred in 205 of the cases reviewed, with 193 (94.1%) cases after LSG and 12 (5.9%) cases following LRYGB. The diagnosis of PMVT occurred within <30 days in most cases.

The common comorbidities among patients diagnosed with PMVT included sleep apnea (50.0%), HTN (36.4%) and dyslipidemia (31.8%). OC use was reported in 45.5% of female patients. History of previous VTE was documented in 15.1% of cases. The hematologic conditions associated with a higher thromboembolic risk were reported in five studies [16,18,21,22,23], with 15 cases positive for thrombophilia.

The presenting symptoms of PMVT cases were detailed in five studies [17,18,21,22,23], with abdominal pain being predominant (96.8%). Diagnosis was established mainly through abdominal CT in 71/73 cases, through magnetic resonance imaging in 2/73 cases and two patients also required diagnostic laparoscopy [17,18,21,22,23,24]. Doppler Ultrasound was utilized for follow-up in 32/38 cases [21,22,23].

Treatment included LMWH in 33/72 cases, UFH in 2/72 cases, a non-specific anticoagulant in 33/72 cases and a supportive treatment in 4/72 cases. Additionally, one patient received endovascular thrombolysis as part of their treatment [16,18,21,22,23]. 

Prophylactic anticoagulation measures were documented in six studies [16,17,18,21,22,23]. LMWH was administered in 73 cases, with one case involving the prior administration of UFH. Additionally, mechanical prophylaxis with compression stockings was reported in 76 cases.

## 4. Discussion

In our case series, all cases of PMVT occurred after LSG, with an incidence of 0,1% and a 30-day mortality rate of 20% (1/5). The mortality occurring over a year post-LSG was not directly attributed to PMVT but to other complications. Thromboembolic events, though rare, are acknowledged as the most common cause of mortality after MBS [26].

The overall rate of VTE and PMVT following MBS is low but its incidence might be underestimated [27]. Our systematic review revealed a PMVT incidence of 0.17%, with 0.24% occurring after LSG and 0.03% after LRYGB. In contrast, a recent meta-analysis reported an incidence of 0.50% [28], but only included LSG. The lower incidence in our review may be attributed to our specific inclusion criteria, which accounted for studies reporting occurrences of PMVT in both LSG and LRYGB. In recent years, LSG has emerged as the most popular procedure for treating obesity [29,30]. This preference is attributed to its perceived advantages, including simplicity, speed and lower invasiveness compared to other MBS, as it only modifies one organ. Studies have reported that LSG is associated with fewer post-surgical complications, including bleeding, serious morbidity, sepsis and reduced rates of 30-day readmission and reoperation [31,32,33]. However, it is crucial for both surgeons and patients to be aware that LSG is associated with a higher incidence of PMVT.

The pathophysiology of PMVT involves one or more components of Virchow’s triad, including reduced portal blood flow, hypercoagulable state, or vascular injury [34]. The potential mechanisms, proposed by Goiten et al. [22], that contribute to the increased risk of PMVT after LSG include altered blood flow post-LSG due to the division of the short gastric vessels, direct physical contact with the SV while working on the omental bursa (which can occur during both LSG and LRYGB), and early postoperative discharge leading to dehydration. Other factors such as a pneumoperitoneum exceeding 14 mmHg, a prolonged reverse Trendelenburg position (duration of surgery) and hypercapnia have also been identified as potential contributors to reduced portal blood flow [35,36].

Regarding other risk factors for PMVT, four cases in our series involved young females with a high BMI and who were taking OC. Comorbidities related to obesity were prevalent among most cases. Notably, PMVT developed despite adequate multi-level, per-protocol, thromboprophylaxis. These findings are consistent with our systematic review results, as 45% of female patients with PMVT reported using OC, a factor that may need further investigation. Carlin et al. [19] identified several independent predictors for PMVT following MBS, including LSG, a prior history of VTE, liver disorders and serious postoperative complications such as obstruction, leaks and hemorrhage.

Hematologic abnormalities, including acquired and inherited thrombophilia, appear to be more prevalent in MBS patients. Parkin et al. [37] reported a thrombophilia prevalence of 52.4% and verified that extended prophylactic anticoagulation reduced the PMVT incidence in MBS patients. This finding suggests that screening for VTE history and hematologic abnormalities before surgery may be warranted, with consideration for extended thromboprophylaxis.

Abdominal pain consistently emerges as the primary presenting symptom in patients with PMVT, as noted by Naymagon et al. [38]. The lack of specificity in abdominal pain underscores the importance of maintaining a high level of clinical awareness to achieve accurate and timely diagnosis. Post-MBS abdominal pain warrants attention from both patients and clinicians to avoid overlooking potential PMVT. The presence of concurrent symptoms such as fever warrants the consideration of septic thrombosis, which affects the treatment and prognosis [39]. Abdominal CT is paramount in the diagnosis of PMVT.

Clinically, PMVT can manifest as either acute or chronic, with no definitive timeframe for differentiation. A potential distinguishing factor is the absence of radiologic findings indicative of chronic PMVT, such as cavernoma or portosystemic collaterals and splenomegaly. In acute PMVT, liver function tests generally remain within normal ranges. Hepatic dysfunction is more likely in patients with prolonged portal hypertension resulting from PMVT [40], but may also arise consequently to multi-organ dysfunction due to septic shock, as observed in one patient described in our case series. The presence of portosystemic collaterals, although variable or inconsistent, may have an important role in the outcome of PMVT.

Most patients with PMVT present early (<30 days) after MBS, suggesting that delayed presentations may represent unrecognized or untreated acute cases, potentially underestimating the true prevalence of PMVT [23]. Early diagnosis, heightened clinical awareness, improved diagnostic techniques and the prompt initiation of anticoagulation therapy have contributed to an improved 5-year survival rate of 85% for acute PMVT [34,40].

The presence of bowel infarction and multi-systemic failure is associated with hospital mortality rates ranging from 20 to 50% [34]. Patients at a high risk of bowel ischemia, infarction and death can be identified by abdominal CT, particularly by observing the progressive extension of thrombus into veins such as the SMV and its smaller tributaries. Additionally, the presence of ascites can indicate increased risk [41]. Although the predictors of survival rates remain unclear, advanced age and thrombosis involving the SMV appear to be significant determining factors [40].

The primary treatment modality for PMVT is anticoagulation, typically subcutaneous LMWH or intravenous UFH, which have shown efficacy in promoting recanalization of the PV/SMV and its branches, reducing the risk of thrombosis progression. In severe PMVT cases, more invasive treatments might be necessary. While the utility of interventional radiology remains somewhat limited, there is growing evidence supporting its efficacy. Mechanical thrombectomy followed by local pharmacologic thrombolysis is recommended to enhance thrombus dissolution [42,43]. Surgical intervention may also be necessary, particularly in instances where bowel segments are compromised [44].

Prophylactic anticoagulation measures, predominantly utilizing LMWH, were documented in six studies, alongside mechanical prophylaxis using compression stockings. Although compression stockings are standard for preventing DVT and PE and are mandatory for patients undergoing MBS, their efficacy in preventing PMVT remains unclear. While guidelines from the ACCP recommend prophylaxis with LMWH, UFH or mechanical prophylaxis with ICP, there is a lack of consensus regarding the standard of care for prophylactic agents, dosing, timing or duration for patients undergoing MBS [5,11,12].

Hasley et al. [45] evaluated the utility of the Caprini risk assessment model for LSG in selecting patients to receive extended courses of prophylaxis and found a low rate of VTE, no PMVT and no bleeding complications. A risk-adjusted approach to VTE prophylaxis in patients undergoing MBS has been proposed by Aminian et al. [46], utilizing their risk calculator. It categorizes patients into moderate, high and very high risk, with an escalating prophylactic approach. For moderate-risk patients, they recommend early mobilization, pneumatic compression and in-hospital prophylaxis. For high-risk patients, 2 weeks of post-discharge prophylaxis is added and for very-high-risk patients, 4 weeks of post-discharge prophylaxis is added [47]. Similarly, the ESA proposed a risk-adjusted approach, defining high-risk patients as those above 55 years and a BMI above 55 kg/m^2^, a history of VTE, venous disease, sleep apnea, hypercoagulability states and pulmonary hypertension [10].

After the treatment of PMVT, it is important to prevent other thromboembolic events. Oral anticoagulation should be maintained for a minimum of 6 months post-discharge [44], considering a more extensive duration based on the thrombosis etiology [43].

There is limited evidence regarding the use of direct oral anticoagulants (DOACs) for prophylaxis and treatment in patients submitted to MBS [48]. While DOACs are used in non-bariatric patients with PMVT [49], their efficacy in MSB patients is controversial due to potential anatomical alterations affecting absorption. DOACs are primarily absorbed in the gastrointestinal tract, particularly the stomach and proximal small intestine [50]. Specifically, Apixaban is absorbed mainly in the small intestine, offering advantages over other anticoagulants as it is unaffected by pH and does not need food restrictions, dose adjustment, monitoring or injections. Its absorption appears unaltered in LSG patients [51]. Surve et al. [52] reported favorable outcomes with thromboprophylaxis with Apixaban for thromboembolic events 30 days post-surgery, independent of the MBS type. While current evidence discourages DOAC use in the acute setting after MBS [53], conclusive data regarding this matter are lacking. Thus, further research is warranted to clarify DOACs’ efficacy and safety profile in this specific population.

Some limitations of this study should be acknowledged. The retrospective nature of most studies and the heterogeneity of the study designs may have introduced bias. The lack of comprehensive information on patient characteristics and risk factors for PMVT impaired a more detailed comparison between the cases of thrombosis post-LSG and post-RYGB, which was the primary objective of this study. This emphasizes the need for standardized reporting in future studies regarding comorbidities and risk factors. Another limitation identified was the accurate determination of the true prevalence of thrombophilia in our results. The diagnostic workup for thrombophilia varies by institution, as it may lead to an underestimation of its actual prevalence. We attempted to conduct a meta-analysis to compare the incidence of PMVT in LSG and LRYGB. However, it was unsuccessful due to numerous studies reporting zero cases of thrombosis post-LRYGB and the limited occurrence of events, rendering statistical analysis unfeasible.

This systematic review provides valuable insights on PMVT incidence following different MBS procedures, particularly noting a higher frequency after LSG. These findings highlight the importance of implementing enhanced prophylactic measures, particularly in patients undergoing LSG with VTE risk factors. It also raises the question of whether patients undergoing LSG should receive prolonged anticoagulants compared to patients submitted for other MBS procedures, namely LRYGB. The lack of specific thromboprophylaxis guidelines for MBS patients emphasizes the need for further research to establish optimal anticoagulant choices, dosing regimens and the duration of prophylaxis for PMVT, tailored to the unique considerations of each MBS patient.

## 5. Conclusions

PMVT is a rare yet potentially life-threatening complication after MBS. According to our case series and systematic review, the occurrence of PMVT appears to be higher in women taking oral contraceptives, those with a higher BMI and following LSG. Given the nonspecific nature of the presenting symptoms, a high level of suspicion is necessary for accurate and timely diagnosis. Treatment involving the prompt administration of anticoagulation is crucial. Notably, no consensus has been reached regarding the standard of care for thromboprophylaxis in MBS patients; therefore, further research on this matter is necessary.

## Figures and Tables

**Table 1:** Characteristics of the patients in our case series.

	Case 1	Case 2	Case 3	Case 4	Case 5
Age	43	23	42	23	32
Sex	Female	Female	Male	Female	Female
BMI (kg/m^2^)	40.4	45.9	39.7	48.2	56.0
Comorbidities	-	Diabetes, asthma	HTN, dyslipidemia, sleep apnea	Sleep apnea	PCOS
Medication	OC	OC, metformin	None	OC	OC
Surgery	LSG	LSG	LSG	LSG	LSG
Thromboprophylaxis	Enoxaparin 60 mg/2 weeks	Enoxaparin 40 mg/2 weeks	Enoxaparin 40 mg/2 weeks	Enoxaparin 40 mg/2 weeks	Enoxaparin 40 mg/4 weeks
Site of thrombosis	SMV, PV	SMV, PV, SV	PV, SV	SMV, PV	SMV, PV
Time until event (days)	11	13	646	16	39
Symptoms/sings	Abdominal pain, fever, leukocytosis, CPR elevation	Abdominal pain, vomit, hematochezia, leukocytosis, CPR elevation	Asymptomatic	Abdominal pain, nausea vomit, fever, leukocytosis	Abdominal pain, nausea, CPR elevation
Treatment	Enoxaparin	UFH	-	UFH, bowel resection	UFH, Alteplase
Post-discharge anticoagulation	Warfarin	-	Warfarin	Enoxaparin	Warfarin
Thrombophilia	Factor II G20210A heterozygote gene mutation	-	None	None	None
Outcome	Partial resolution	Deceased	Chronic Thrombosis	Deceased (1 year later)	Partial resolution

CPR, C-reactive protein; HTN, hypertension; LSG, Laparoscopic Sleeve Gastrectomy; OC, oral contraceptive; PCOS, polycystic ovarian syndrome; PV, portal vein; SMV, superior mesenteric vein; SV, splenic vein; UFH, unfractionated heparin.

**Table 2:** Baseline characteristics of the included studies.

**Hospital Stay PMVT (SD)**	**LRYGB**	N/A	1.0 (1.0)	N/A	N/A	N/A	N/A	2.0 (0.5)	3.0 (2.0–6.0)	N/A	N/A
	**LSG**	N/A	1.0 (0)	N/A	N/A	N/A	N/A			N/A	N/A
**Operative Time PMVT (SD)**	**LRYGB**	N/A	91.0 (0)	N/A	N/A	N/A	64.8 (27.6)	N/A	85.0 (53–0-152.0)	N/A	N/A
	**LSG**	N/A	45.0(6.7)	N/A	N/A	N/A		N/A		N/A	N/A
**BMI PMVT (SD)**	**LRYGB**	N/A	39.0 (0)	N/A	N/A	N/A	44.6 (5.3)	0	0	N/A	N/A
	**LSG**	N/A	37.2(1.2)	N/A	N/A	N/A		44.3 (3.9)	41.8 (4.5)	N/A	N/A
**Age PMVT (SD)**	**LRYGB**	N/A	29.0 (0)	N/A	N/A	N/A	N/A	0	0	N/A	N/A
	**LSG**	N/A	35.4(3.6)	N/A	N/A	N/A	N/A	38.0 (9.9)	35.1 (10.5)	N/A	N/A
**Female PMVT (%)**	**LRYGB**	N/A	0	N/A	N/A	N/A	53.84	0	0	N/A	N/A
	**LSG**	N/A	100.00	N/A	N/A	N/A		41.20	56.00	N/A	N/A
**Hospital Stay (SD)**	**LRYGB**	2.7 (1.5)	N/A	N/A	3.4 (4.4)	N/A	N/A	2.8	N/A	N/A	N/A
	**LSG**	2.4 (1.2)	N/A	N/A	2.8 (0.6)	N/A	N/A		N/A	5 (4–7)	N/A
**Operative Time (SD)**	**LRYGB**	154.0 (19.1)	N/A	N/A	106.2 (33.2)	89.3	N/A	N/A	N/A	N/A	N/A
	**LSG**	107.9 (20.6)	N/A	N/A	76.6 (28.0)		N/A	N/A	N/A	75.0 (60.0–105.0)	N/A
**BMI (SD)**	**LRYGB**	45.1 (7.5)	N/A	47.9 (5.8)	38.0 (3.4)	47.7	N/A	44.4	N/A	N/A	47.7 (38.2–57.2)
	**LSG**	42.3 (6.6)	N/A	46.6 (6.3)	37.90 (4.6)		N/A		N/A	44.5 (39.3–49.8)	
**Age (SD)**	**LRYGB**	41.9 (10.3)	N/A	43.4 (7.6)	37.0 (10.3)	45.5	N/A	45.0	N/A	N/A	45.0 (21.0–59.0)
	**LSG**	36.4 (12.7)	N/A	40.4 (7.1)	36.4 (11.7)		N/A		N/A	40.0 (35.0–39.0	
**Female (%** **)**	**LRYGB**	46.30	N/A	78.00	76.60	79.00	N/A	N/A	N/A	N/A	83.50
	**LSG**	60.00	N/A	66.00	76.20		N/A	N/A	N/A	70.00	
**PMVT**	**LRYBG**	0	1	0	1	7	2	0	0	0	1
	**LSG**	4	5	10	9	109	22	16	16	2	0
**Surgery**	**LRYGB**	175	N/A	45	786	32,009	N/A	966	762	120	666
	**LSG**	400	N/A	84	811	59,462	N/A	4355	2886	1192	148
**N**		575	N/A	129	1597	102,869	26	5706	4386	1400	
**Design**		Retrospective cohort	Case series	Prospective cohort	Retrospective case-control	Prospective cohort	Cross sectional	Retrospective cohort	Retrospective cohort	Retrospective cohort	Retrospective cohort
**Country**		Lebanon	Brazil	Egypt	Chile	US	Italy	Israel	Israel	Poland	Canada
**Study Period**		01/2008–12/2013	N/A	12/2014–03/2018	01/2006–09/2009	06/2006–11/2021	N/A	01/2007–06/2012	01/2006–11/2015	02/2014–03/2018	07/2009–12/2012
**Year**		2017	2020	2020	2012	2022	2021	2013	2016	2020	2018
**Author**		Aridi, et al. [15]	Barros, et al. [21]	Bassiouny, et al. [24]	Boza, et al. [16]	Carlin, et al. [19]	Carrano, et al. [18]	Goitein, et al. [22]	Rottenstreich, et al. [23]	Wysocki, et al. [25]	Tseng, et al. [17]

N/A, not applicable.

**Table 3:** Characteristics of patients with PMVT.

Variable	N	(%)
Female	33/62	53.33
Diabetes	3/22	13.6
Dyslipidemia	44/138	31.8
HTN	8/22	36.4
Sleep apnea	3/6	50.0
Liver disorders	29/138	21.0
History of VTE	26/172	15.1
Thrombophilia	15/72	20.8
FVLM	5	
FVIIIE	4	
PSD	4	
PCD	2	
FIIG20210AM	1	
MTHFRD	1	
JAK2M	1	
NSTS	1	
OC	10/22	45.5
Symptoms		
Abdominal pain	61/63	96.8
Fever	16/56	28.6
Nausea	30/62	48.4
Vomit	26/62	41.9
Asymptomatic	2/16	12.5
Time until event		
<30 days	182/203	89.7
>30 days	21/203	10.3

FIIG20210AM, Factor II G20210A gene mutation; FVLM, Factor V Leiden mutation; FVIIIE, Factor VIII elevation; HTN, hypertension; JAK2M, JAK2 mutation; MTHFRD, Methylenetetrahydrofolate reductase deficiency; NSTS, non-specific prothrombotic state OC, oral contraceptive; PSD, Protein S deficiency; PCD, Protein C deficiency.

## Data Availability

The data for this study are based on the published literature and are available upon request from the corresponding author.

## Supplementary Materials

The following supporting information can be downloaded at: https://www.mdpi.com/article/10.3390/jpm14070722/s1, Table S1: Search terms and queries; Table S2: Description of the answers in NHLBI quality assessment criteria tool for Observational Cohort and Cross-Sectional Studies. N/A, not applicable, NR, not reported; Table S3: Description of the answers in NHLBI quality assessment criteria tool for Case-Control Studies. CD, cannot determine; Table S4: Description of the answers in NHLBI quality assessment criteria tool for Case Series Studies. N/A, not applicable.
