# Supplementary material for: Portomesenteric Venous Thrombosis after Bariatric Surgery: A Case Series and Systematic Review Comparing LSG and LRYGB
Source: J Pers Med. 2024 Jul 4;14(7):722. doi: 10.3390/jpm14070722 (PMC11277930; doi:10.3390/jpm14070722)
Supplement: Supplementary file 1 [file jpm-14-00722-s001.zip › SI.pdf]

Supplementary Information

Supplementary Table S1. – Search terms and queries.

	**Keywords**
**PubMed**	("Obesity/surgery"[Mesh] OR "Bariatric Surgery"[Mesh] OR "bariatric surger*"[Title/Abstract] OR "sleeve gastrectomy"[Title/Abstract] OR "gastric bypass"[Title/Abstract] OR "laparoscopic sleeve gastrectomy"[Title/Abstract] OR "laparoscopic gastric bypass"[Title/Abstract]) AND ("Venous Thrombosis"[Mesh] OR "Mesenteric Ischemia"[Mesh] OR "mesenteric vein"[Title/Abstract] OR "portal vein"[Title/Abstract] OR "portomesenteric vein thrombosis"[Title/Abstract] OR "vein thrombosis"[Title/Abstract])
**Web of Science**	TS=(bariatric surgery OR sleeve gastrectomy OR gastric bypass OR laparoscopic sleeve gastrectomy OR laparoscopic gastric bypass) AND TS=(mesenteric vein thrombosis OR portal vein thrombosis OR portomesenteric vein thrombosis OR mesenteric ischemia, OR venous thrombosis)
**Scopus**	TITLE-ABS-KEY(("bariatric surgery" OR "sleeve gastrectomy" OR "gastric bypass" OR "laparoscopic sleeve gastrectomy" OR "laparoscopic gastric bypass") AND ("mesenteric vein thrombosis" OR "portal vein thrombosis" OR "portomesenteric vein thrombosis" OR "mesenteric ischemia" OR "venous thrombosis"))

Supplementary Table S2. – Description of the answers in NHLBI quality assessment criteria tool for Observational Cohort and Cross-Sectional Studies

	**Aridi et al.**	**Bassiouny, et al.**	**Carlin et al.**	**Carrano et al.**	**Goitein, et al.**	**Naymagon et al.**	**Rottenstreich, et al**	**Wysocki, et al.**	**Tseng et al.**
**1.**	YES	YES	YES	YES	YES	YES	YES	YES	YES
**2.**	YES	YES	YES	YES	YES	YES	YES	YES	YES
**3.**	YES	YES	YES	NO	YES	YES	YES	YES	YES
**4.**	YES	YES	YES	YES	YES	YES	YES	YES	YES
**5.**	NO	NO	NO	NO	NO	NO	NO	NO	NO
**6.**	YES	YES	YES	NO	YES	YES	YES	YES	YES
**7.**	YES	YES	YES	NO	YES	YES	YES	YES	YES
**8.**	N/A	N/A	N/A	N/A	N/A	N/A	N/A	N/A	YES
**9.**	YES	YES	YES	YES	YES	YES	YES	YES	YES
**10.**	N/A	N/A	N/A	N/A	N/A	N/A	N/A	N/A	YES
**11.**	YES		YES	YES	YES	NO	YES	YES	YES
**12.**	NO	NO	NO	NO	NO	NO	NO	NO	NO
**13.**	NO	NR	NR	N/A	NR	NR	NR	YES	NO
**14.**	YES	YES	YES	NO	YES	YES	YES	YES	NO
**YES/NO**	9/3	8/2	9/2	5/6	9/2	8/4	9/2	10/2	10/4

1 - Was the research question or objective in this paper clearly stated?

2 – Was the study population clearly specified and defined?

3 – Was the participation rate of eligible persons at least 50%?

4 – Were all the subjects selected or recruited from similar populations? Were inclusion and exclusion criteria for being in the study prespecified and applied uniformly to all participants?

5 - Was a sample size justification, power description, or variance and effect estimates provided?
6 - For the analyses in this paper, were the exposure(s) of interest measured prior to the outcome(s) being measured?
7 - Was the timeframe sufficient so that one could reasonably expect to see an association between exposure and outcome if it existed?
8 - For exposures that can vary in amount or level, did the study examine different levels of the exposure as related to the outcome?
9 - Were the exposure measures (independent variables) clearly defined, valid, reliable, and implemented consistently across all study participants?
10 - Was the exposure(s) assessed more than once over time?
11 - Were the outcome measures (dependent variables) clearly defined, valid, reliable, and implemented consistently across all study participants?
12 - Were the outcome assessors blinded to the exposure status of participants?
13 - Was loss to follow-up after baseline 20% or less?
14 - Were key potential confounding variables measured and adjusted statistically for their impact on the relationship between exposure(s) and outcome(s)?

Supplementary Table S3. – Description of the answers in NHLBI quality assessment criteria tool for Case-Control Studies

	**Boza et al.**
**1 - Was the research question or objective in this paper clearly stated and appropriate?YES**	YES
**2 – Was the study population clearly specified and defined?**	YES
**3 – Did the authors include a sample size justification?**	NO
**4 – Were controls selected or recruited from the same or similar population that gave rise to the cases (including the same timeframe)?**	YES
**5 - Were the definitions, inclusion and exclusion criteria, algorithms or processes used to identify or select cases and controls valid, reliable, and implemented consistently across all study participants?**	YES
**6 - Were the cases clearly defined and differentiated from controls?**	YES
**7 - If less than 100 percent of eligible cases and/or controls were selected for the study, were the cases and/or controls randomly selected from those eligible?**	CD
**8 - Was there use of concurrent controls?**	NO
**9 - Were the investigators able to confirm that the exposure/risk occurred prior to the development of the condition or event that defined a participant as a case?**	YES
**10 - Were the measures of exposure/risk clearly defined, valid, reliable, and implemented consistently (including the same time period) across all study participants?**	YES

**11 - Were the assessors of exposure/risk blinded to the case or control status of participants?**	NO
**12 - Were key potential confounding variables measured and adjusted statistically in the analyses? If matching was used, did the investigators account for matching during study analysis?**	YES
**YES/NO**	8/3

Supplementary Table S4. – Description of the answers in NHLBI quality assessment criteria tool for Case Series Studies.

	**Barros et al.**
**1 – Was the study question or objective clearly stated?**	YES
**2 – Was the study population clearly and fully described, including a case definition?**	YES
**3 – Were the cases consecutive?**	NO
**4 – Were the subjects comparable?**	YES
**5 - Was the intervention clearly described?**	YES
**6 - Were the outcome measures clearly defined, valid, reliable, and implemented consistently across all study participants?**	YES
**7 - Was the length of follow-up adequate?**	YES
**8 - Were the statistical methods well-described?**	N/A
**9 - Were the results well-described?**	YES
**YES/NO**	7/1
